# Electroencephalography (EEG) for Neurological Prognostication in Post-Anoxic Coma Following Cardiac Arrest and Its Relationship to Outcome

**DOI:** 10.3390/brainsci14121264

**Published:** 2024-12-17

**Authors:** Zaitoon Shivji, Nathaniel Bendahan, Carter McInnis, Timothy Woodford, Michael Einspenner, Lisa Calder, Lysa Boissé Lomax, Garima Shukla, Gavin P. Winston

**Affiliations:** 1 EEG Department, Kingston Health Science Center, Kingston, ON K7L 2V7, Canada; zaitoon.shivji@kingstonhsc.ca (Z.S.);; 2 Edmond J. Safra Program in Parkinson’s Disease, Division of Neurology, Toronto Western Hospital, University Health Network, Toronto, ON M5T 2S8, Canada; 3 Department of Medicine, University of Toronto, Toronto, ON M5S 1A1, Canada; 4 Department of Medicine, University of Calgary, Calgary, AB T2N 4Z6, Canada; 5 Department of Medicine, Division of Neurology, Queen’s University, Kingston, ON K7L 3N6, Canada; 6 Centre for Neuroscience Studies, Queen’s University, Kingston, ON K7L 3N6, Canada

**Keywords:** cardiac arrest, electroencephalography, anoxic coma, prognosis

## Abstract

Background/Objectives: Cardiac arrest may cause significant hypoxic–ischemic injury leading to coma, seizures, myoclonic jerks, or status epilepticus. Mortality is high, but accurate prognostication is challenging. A multimodal approach is employed, in which electroencephalography (EEG) forms a key part with several recognised patterns of prognostic significance. Methods: In this retrospective study, clinical and qualitative features of the EEG of patients admitted to the Intensive Care Unit (ICU) at Kingston General Hospital following cardiac arrest from 2017 to 2020 were reviewed. The study included 81 adult patients (≥18 years). Outcome was assessed using the Cerebral Performance Category (CPC) as 1–2 (favourable) or 3–5 (unfavourable). EEG patterns were divided into groups within the highly malignant, malignant and benign patterns described in the literature. Results: There were a wide range of causes and 22% had a favourable outcome. Highly malignant, malignant and benign patterns were associated with survival in 0%, 70% and 100%, respectively, and favourable outcomes in 0%, 48% and 100%. All patients with seizures died, and 94% with myoclonus had unfavourable outcomes. In contrast, EEG reactivity and improvement on follow-up EEG were associated with a favourable outcome. Conclusions: Highly malignant EEG, seizures and myoclonus were associated with unfavourable outcomes, while patients with malignant EEG had better outcomes.

## 1. Introduction

Cardiac arrest is common and may result in significant hypoxic–ischemic injury. According to the American Heart Association, there are more than 356,000 out-of-hospital cardiac arrests annually in the US [1,2,3]. Although resuscitation practices have greatly improved over the years, the outcome remains poor [3], with survival from out-of-hospital cardiac arrest remaining under 10% [1,2]. Even when patients are resuscitated in the hospital, fewer than 20% of patients are discharged home [4,5,6].

By the time cardiopulmonary resuscitation (CPR) restores adequate perfusion, the brain may have been critically injured [3], and this risk is greater with longer periods of hypoxia. Neurological recovery sufficient to lead an independent life occurs in only a small proportion of patients, whilst others are left with significant neurological disability. Hypoxic–ischemic encephalopathy may lead to coma, a vegetative state, seizures, myoclonic jerks, or status epilepticus. Patients who awake from coma generally do so within 3 days after CPR, or significant neurologic impairment can be expected [3,7]. The prognosis of such patients with post-anoxic coma is a challenging task for physicians.

Prediction of neurological outcomes after resuscitation from cardiac arrest is an important component of the management of comatose resuscitated patients. Early and accurate identification of patients with an expected favourable neurological recovery is beneficial [3,8,9] and allows discussions with families around withdrawal of life-sustaining treatment for those with poor chances of outcome. Clinical examination features such as absent pupillary light response or corneal reflexes, extensor or no motor response to pain after 3 days of assessment, and myoclonic status epilepticus combined with electrophysiological procedures can identify recognised markers of poor outcome after CPR [3,7,10].

Electroencephalography (EEG), a non-invasive technique to monitor neuronal activity, is widely used to assess neurological status after cardiac arrest, both as a diagnostic and prognostic tool and may reveal subclinical seizures. Both EEG and somatosensory evoked potentials (SSEP) [3,10,11,12,13,14,15] can be highly specific for differentiating between poor versus favourable prognoses if they are applied to appropriate patient populations, as there are recognised patterns with prognostic significance. However, they should not be considered in isolation but in combination with other assessments. A multimodal approach to prognostication, that includes the use of EEG, may particularly improve early prediction of clinical evolution after cardiac arrest [3,10,11,12,13,14,15].

Over the years, many EEG scoring categories or grading have been used. An initial grading system for prognostication after cardiac arrest was published with five different grades (I–V) based on the dominant frequency or presence of abnormal patterns [9,16,17,18]. Subsequently, in 1970 [19], the effects of the reactivity of the EEG to stimuli were added, increasing the diagnostic accuracy of the EEG grading scale.

More recently, the American Society of Clinical Neurophysiology (ACNS) provided standardised critical care EEG terminology to include a classification for the background and other relevant patterns, initially in 2012 [20] and revised in 2021 [21]. This new terminology maximises interrater reliability [15,22,23,24,25]. Using these guidelines, three categories of EEG patterns to reliably predict outcomes in comatose patients following cardiac arrest (highly malignant, malignant and benign) have been proposed [26] and validated [15]:

**Highly malignant**

- Suppressed background without discharges.
- Suppressed background with continuous periodic discharges.
- Burst–suppression background (with or without discharges).

**Malignant EEG**

- Periodic or rhythmic patterns (abundant periodic discharges; abundant rhythmic polyspike-/spike-/sharp-and-wave; unequivocal electrographic seizure).
- Malignant background (discontinuous background; low voltage background; reversed anterior–posterior gradient).
- Reactivity (absence of background reactivity or only stimulus-induced discharges).

**Benign EEG**

- Absence of all malignant features stated above.

Subsequent work has shown the importance of these categories and early EEG evaluation for prognosis in patients following cardiac arrest [25,27].

The aim of the present study is to retrospectively evaluate the patterns seen on EEG in patients with post-anoxic coma following cardiac arrest in a single centre and their relationship to outcome, evaluating in the context of the existing literature on EEG patterns in this clinical situation. We chose to focus solely on EEG findings but acknowledge that this forms only part of a multimodal prognostic approach.

## 2. Materials and Methods

### 2.1. Patient Cohort and Outcome Assessment

We retrospectively reviewed medical records and EEG recordings of patients with post-anoxic coma due to cardiac arrest admitted to the ICU at Kingston General Hospital, Ontario, Canada. We included all adult patients (18 years or over) from 2017 to 2020 who were referred for EEG. Data extracted included etiology, age, gender, seizures, myoclonus, sedation with propofol, therapeutic hypothermia (TH), imaging findings (cortical and subcortical injuries or both) and outcome.

The targeted body temperature for TH is usually 33–36 degrees Celsius [28]. Highly malignant and malignant patterns may occur during the initial period after resuscitation and during the target temperature management among survivors who have shown good outcomes [12]. Thus, EEGs recorded during TH were replaced by subsequent EEGs after rewarming to a temperature of >36 degrees Celsius where available.

Patient outcome was assessed at a follow-up rehabilitation appointment within 3–6 months (for those who did not die during or shortly after admission) using the Cerebral Performance Category (CPC) where 1 is a normal or mild disability, 2 is a moderate disability (but independent), 3 is a severe disability, 4 is unconscious (coma/vegetative state) and 5 is brain death [6,24]. We dichotomised outcomes into CPC 1–2 (favourable) and CPC 3–5 (unfavourable) [27,29,30]. 

Ethics approval for retrospective use of clinically acquired data was obtained from the Queen’s University Health Sciences and Affiliated Teaching Hospitals Research Ethics Board (DMED-2413-20).

### 2.2. Analysis of EEG Recordings

Patients underwent routine digital EEG recordings for at least 20 min with 23 scalp electrodes placed according to the 10–20 International System of electrode placement, reformatted to both bipolar and referential montages. The filter settings were 0.5 Hz and 70 Hz. Sedation was stopped prior to all recordings.

Data extracted from a qualitative review of EEG included background activity, presence of myoclonus and/or seizures during EEG, presence of rhythmic patterns or periodic patterns and reactivity of EEG upon stimulation. All EEG recordings were interpreted by board-certified epileptologists.

Reactivity of the EEG was defined as distinct changes in amplitude or frequency or attenuation of background following external stimuli. Stimuli were tactile or nociceptive stimulation, auditory stimuli (clapping and voice sounds) or passive eye-opening and suctioning for deep stimulation. An increase in muscle artifacts and or movement during stimulation was not considered reactivity [8,20,25].

A single rater classified recordings using the categories described previously (highly malignant, malignant and benign) based on ACNS terminology [15], including a review of the original recording, but did not include reactivity as a separate category as there were no recordings demonstrating a lack of reactivity that were not already classified as highly malignant or malignant. We instead quote the proportion in each category that shows reactivity. Where several EEG recordings existed in the same patient, we also evaluated the change over time from the initial EEG pattern and its relationship to the outcome.

### 2.3. Neuroimaging Findings

We reviewed the neuroimaging findings based on the reports and classified them into 5 categories. Classical findings of hypoxic–ischemic injury include loss of grey/white matter differentiation and oedema on computed tomography (CT) or diffusion restriction on magnetic resonance imaging (MRI) [31]. We divided these findings into diffuse hypoxic injuries where changes were widespread (e.g., multiple cortical areas, cortex and basal ganglia) and limited when present in a single cortical or subcortical area. Other findings included acute ischemic changes (e.g., stroke), haemorrhage (both intraparenchymal and subdural) and unremarkable (including incidental findings such as atrophy or microvascular disease). We used MRI findings where available and, if not, a CT scan.

## 3. Results

### 3.1. Characteristics of Cohort

A total of 81 patients were identified for the study, with a time delay from cardiac arrest to referral for EEG recording varying from 2 to 14 days. The final cohort was 81 patients (57 males, 24 females, aged 18 to 87 years, Table 1), in whom 18 (22%) of the patients had a favourable outcome (CPC 1–2), whilst 63 (78%) had unfavourable outcomes (CPC 3–5).

Causes of cardiac arrest, as stated in the clinical charts, included ventricular fibrillation, myocardial infarction, pulseless electrical activity, respiratory arrest, airway obstruction, pulmonary embolism, sepsis, aortic dissection and haemorrhagic stroke (Table 2). The indications given for EEG referral included decreased level of consciousness, myoclonic jerks or twitching post-cardiac arrest, possible seizure post-cardiac arrest and agitation/confusion (Table 3).

Only a single patient who had highly malignant EEG during TH and warming did not have a follow-up EEG and had an outcome of CPC 5 (death).

### 3.2. General EEG Features and Outcomes

**Figure 1:**
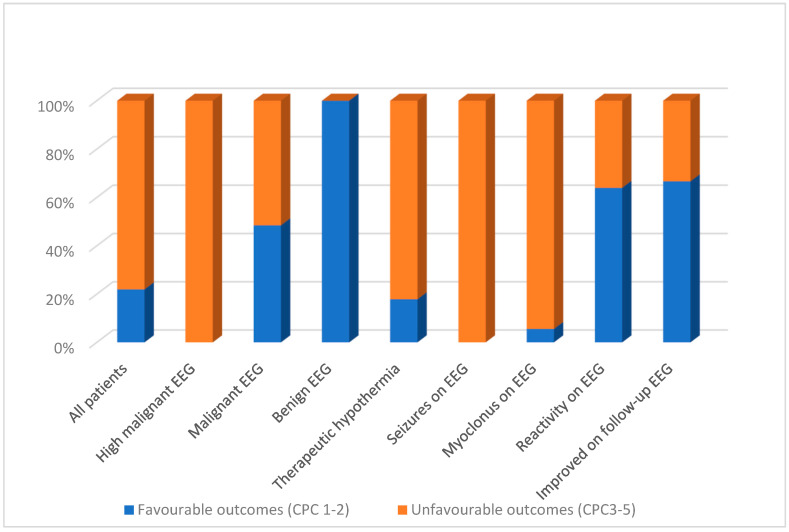
Proportion of favourable vs. unfavourable outcomes (based on CPC) and the relationship to clinical variables derived primarily from EEG.

General EEG features and outcomes are noted in Table 1 and Figure 1. None of the patients who had seizures or were categorised as highly malignant on EEG survived. For those with myoclonus, only a single patient survived (1 of 18, 6%), but most of these patients also had a highly malignant EEG pattern. 

Reactivity was seen on the EEG in 25/80 (31%) of the patients (one patient was not tested, and one showed stimulus-induced rhythmic, periodic or ictal discharges (SIRPIDs)). Most patients (72%) with reactive EEG survived, and the majority (62%) had a favourable outcome.

Of the 40 patients who had follow-up EEG requested as clinically indicated, 9 (23%) showed improvement, as detailed below. All patients demonstrating improvement on EEG survived, and most (67%) had a favourable outcome.

### 3.3. Specific EEG Patterns and Outcomes

**Figure 2:**
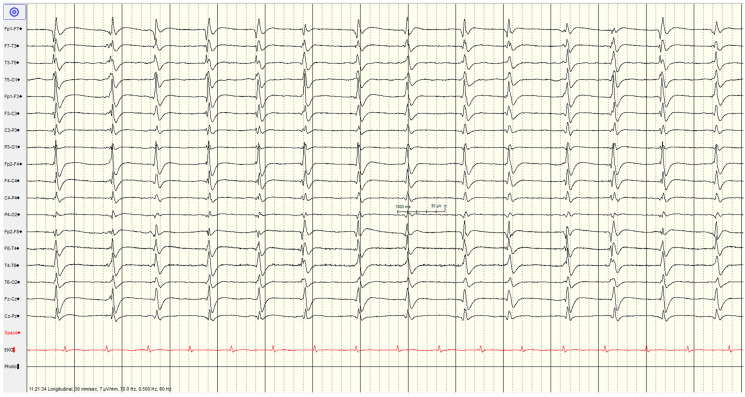
Example of highly malignant EEG pattern—suppressed background with continuous periodic discharges.

**Figure 3:**
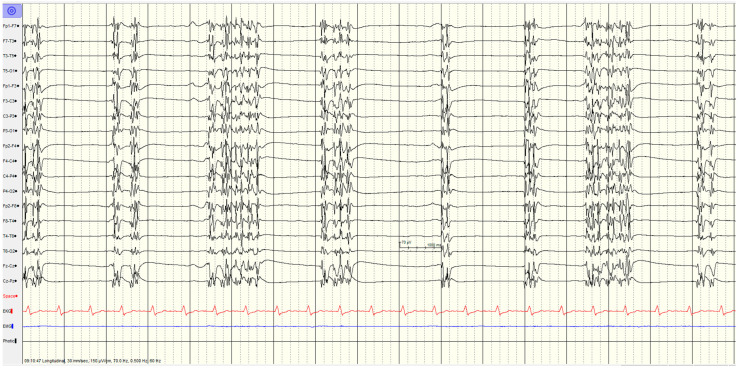
Example of highly malignant EEG pattern—burst–suppression background with or without discharges.

**Figure 4:**
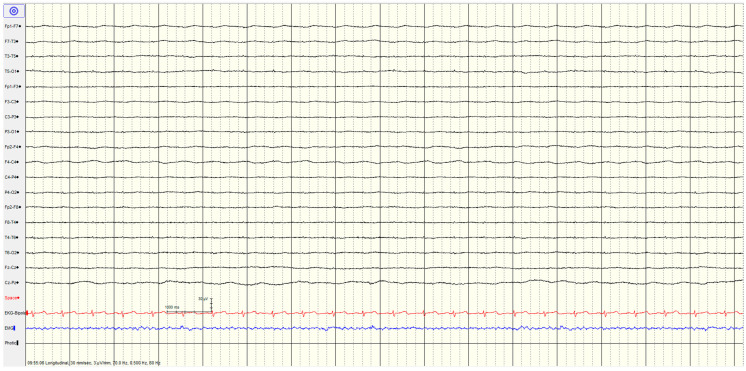
Example of highly malignant EEG pattern—suppressed background without discharges (sensitivity is 3 μV/mm).

**Figure 5:**
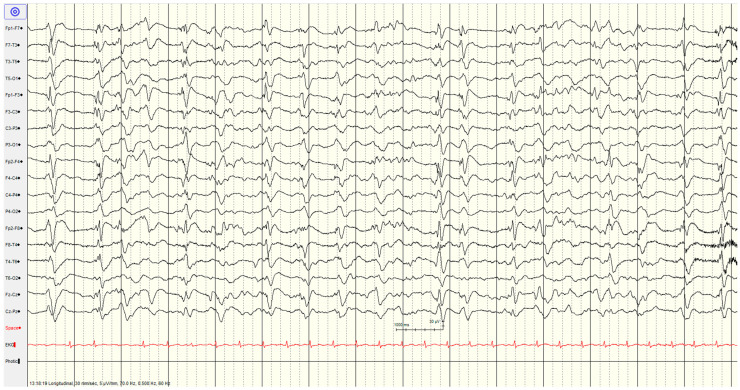
Example of malignant EEG pattern—periodic or rhythmic patterns, showing abundant generalised periodic discharges (GPDs).

**Figure 6:**
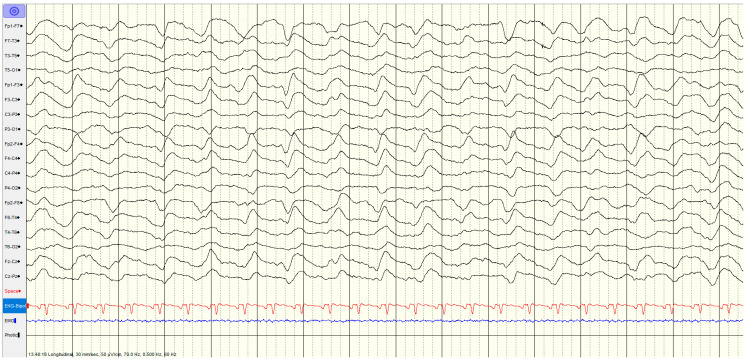
Example of malignant EEG pattern—malignant background, showing reversed anterior–posterior gradient.

The specific EEG patterns classified into highly malignant (Figure 2, Figure 3 and Figure 4), malignant (Figure 5 and Figure 6) and benign and their relationship to outcomes are noted in Table 4 and Figure 1.

#### 3.3.1. High Malignant EEG Pattern

Overall, 46 (57%) of the patients showed a **highly malignant** EEG pattern (Figure 2, Figure 3 and Figure 4). None of these patients showed reactivity on EEG or improvement on a subsequent EEG when performed (from day 1 to 10), and none in this category survived.

The most common pattern, representing 23 patients, was a suppressed background with continuous periodic discharges, of whom around two-thirds also had seizures or myoclonus, and one patient demonstrated SIRPIDs (which showed generalised discharges and seizures). Most (12/22, 55%) had diffuse hypoxic changes on imaging, whereas the remainder showed more limited hypoxic changes (*n* = 5), acute ischemic changes (*n* = 2) or were unremarkable (*n* = 3).

In the category of burst–suppression background with or without the generalised spike- and slow-wave discharges, the recordings with a burst–suppression background with generalised spike- and slow-wave discharges, all patients had seizures or myoclonus, whereas only a single patient without spike-wave discharges had seizures. Just over half (5/9, 56%) had diffuse hypoxic changes on imaging, with the other 4 scans being unremarkable.

A suppressed background (<10 μV) without discharges was not associated with seizures or myoclonus. Most (9/10, 90%) had diffuse hypoxic changes on imaging, with the last having only ischemic changes.

#### 3.3.2. Malignant EEG Pattern

A total of 33 (41%) had a **malignant** EEG pattern (Figure 5 and Figure 6). Overall survival in this category was 70%, although only 48% had a favourable outcome. The proportion of survival was highly significantly different between highly malignant and malignant groups (χ^2^ = 127.373, *p* < 0.001), as was the proportion with favourable outcomes (χ^2^ = 109.675, *p* < 0.001).

In the first malignant category pattern of abundant periodic discharges or rhythmic patterns (polyspike-/spike-/sharp-and-wave), there were 11 patients. Four of the EEGs showed generalised as well as lateralised discharges; some showed lateralised periodic discharges. A single patient with sharps at the vertex, associated with myoclonus, survived. Most patients in this category demonstrated reactivity (64%), and the majority who had follow-up EEG (time range 1–4 days) demonstrated improvement (71%). Improvements included one patient who developed sleep architecture, three patients with improved background activity and a final patient with decreased slowing and sharp activity. Only two patients showed an increase in slowing or burst suppression patterns. Overall favourable outcome in this subcategory was 36%. Only 2/11 (18%) had diffuse hypoxic changes on imaging, with the rest demonstrating more limited hypoxic changes (*n* = 1), ischemic changes (*n* = 4), haemorrhage (*n* = 1) or being unremarkable (*n* = 3).

In the second subcategory of the malignant pattern with discontinuous background, low voltage background and reversed anterior–posterior gradient, the majority showed reactivity (73%), and when repeated (after 1–4 days), 60% showed an improvement in background activity. The others showed an increase in slowing or lower amplitude EEG. There was 82% survival in this group with a favourable outcome (CPC1–2) in 55%. The EEG in this group did not show epileptic activity (spike/sharp- and slow-wave pattern). Few (3/22, 14%) had diffuse hypoxic changes, with the rest showing more limited hypoxic changes (*n* = 1), ischemic changes (*n* = 5), haemorrhage (*n* = 3) or being unremarkable (*n* = 10).

#### 3.3.3. Benign EEG Pattern

The remaining two patients (2%) showed a **benign** EEG pattern (absence of all malignant features). All showed reactivity, and all survived with favourable outcomes. Neuroimaging studies were unremarkable.

## 4. Discussion

### 4.1. Main Findings of the Study

We systematically report the EEG findings and their relationship to outcome in a single-centre cohort of patients with post-anoxic coma following cardiac arrest. In line with prior literature, we found that most patients (79%) had an unfavourable outcome. A highly malignant pattern, seizures and myoclonus on EEG recordings were each highly associated with unfavourable outcomes, whereas patients with EEG reactivity or demonstrating improvement on follow-up EEG were more likely to have a favourable outcome.

### 4.2. Role of EEG in Prognostication

EEG has developed significantly as an important tool since the initial important observations regarding EEG abnormalities, made by Gibbs, Gibbs and Lennox, in comatose and altered conscious state patients [28]. Many EEG grading processes have since been suggested including Hockaday and colleagues in 1965 [16].

The ACNS established standardised terminology and criteria for interpreting critical care EEGs in 2012 [20] that have since been updated in 2021 [21]. For potential use in prognostication, the consistent use of EEG terminology is essential.

We used the 2012 criteria since the EEGs were recorded and interpreted prior to the publication of the newer guidelines, and the definitions of highly malignant and malignant are based on this. However, the changes made in 2021 do not affect the definitions we use.

### 4.3. EEG Background

EEG background activity can help to predict favourable outcomes in patients after cardiac arrest [32]. The voltage of background EEG is defined as low when most or all activity is <20 μV (measured from peak to peak) in a longitudinal bipolar montage with standard 10–20 electrodes, while suppression is defined as all voltage being <10 μV [8,12]. The amplitude of the EEG signal may depend on the effect of drugs, body temperature and a variety of technical conditions such as electrode–scalp impedance, inter-electrode distances, type and placement of the electrodes and type of filters adopted. 

The burst suppression EEG pattern is alternate bursts of EEG activity of variable duration and amplitude alternate with periods of suppression or flattening. Bursts consist of high amplitude slow activity, spike-and-wave or polyspike- and slow-wave complexes that are epileptic [11]. In cardiac arrest, the physiology is not clear, but it is generally accepted that burst suppression occurs from “dissociation of the cortex from the intrinsic pacemaker of neurons in the reticular thalamus” [11]. This indicates severe injury to the thalamus, cerebral cortex and the interconnecting relay circuits [11,15]. This pattern is usually noted when there is severe cortical damage [23].

Low amplitude or burst suppression patterns observed after an anoxic episode may be considered reversible in the absence of sedation [24]. Although EEG may be transiently suppressed early on after cardiac arrest, it may subsequently recover with dynamic changes seen in EEG patterns after cardiac arrest [11,13]. Persistence of suppression after 24–72 h or non-reactivity is associated with unfavourable outcomes [24]. Thus, for predictive purposes, the timing of EEG recording is important [24]. In our study, EEG recordings were primarily recorded beyond this initial period based on the referral pattern.

These EEG patterns are considered part of the highly malignant category, especially burst suppression with identical (monomorphic) bursts of spike-and-wave or sharps, which is highly correlated with severe anoxic brain injury and specific for poor outcomes [15,27]. Overall, most patients with highly malignant EEG patterns (including suppressed background without discharges, suppressed background with continuous periodic discharges, or burst–suppression background with or without discharges) have poor outcomes [11,12,15,17,33]. In our study, no patient in this category survived.

### 4.4. EEG Reactivity

EEG patterns showing reactivity and continuity seem promising as prognostic indicators [14,19,24,25,28]. EEG reactivity was tested by assessing reproducible changes in amplitude or frequency of EEG background following external stimuli, such as tactile or nociceptive stimulation, auditory stimuli (clapping and voice sounds) or visual, passive eye-opening in stepwise manner and noxious stimuli like sternum pressure or rub. These stimuli should be at short intervals to see the effect of stimuli, if any [8,12,19]. Favourable outcomes can still be seen with the absence of EEG reactivity, although the outcome is generally worse with status myoclonus [8]. Importantly, movement and muscle artifacts cannot be marked as reactive EEG [8,15,25]. In our cohort, nearly all patients with reactivity survived, and most (64%) had favourable outcomes.

### 4.5. Interictal Epileptiform Activity

Periodic epileptic discharges are defined as repetitive monotonous sharp transients occurring throughout the recording, typically every 1 to 3 s, with some irregularity in the interval between discharges, without entraining into discrete electrographic seizures. Periodic discharges may be lateralised (LPDs), bilateral independent (BIPDs), generalised (GPDs) or multifocal [12,20]. Generalised periodic discharges (GPDs) require the occurrence of periodic complexes occupying at least 50% of a standard 20 min EEG recording over both hemispheres in a symmetric, diffuse and synchronised manner [12], giving the appearance of a burst suppression pattern. 

Patients whose EEG shows epileptiform activity with a continuous background may be effectively treated with anti-seizure medications (ASM) [8]. However, the difficulty is distinguishing GPDs that are treatable with anti-seizure drugs from severe ischemic damage. Recommendations are that the EEG in these patients need to be evaluated regularly over time rather than just recorded once. Frequency and timing of follow-up EEG would depend on the EEG findings along with clinical correlation.

### 4.6. Seizures

Seizures and myoclonus are associated with poor outcomes [24,34,35]. When seizures occur early during the first few days, they are usually associated with unfavourable features like unreactive or suppressed EEG background [14,33]. EEG is also important for detecting subclinical, non-convulsive seizures. Seizures can be missed, especially in patients who are sedated [15,35]. Timing and duration of EEG monitoring are important, especially if the patient is undergoing TH or is on sedative treatment.

Myoclonus is sudden, brief shock-like muscle contractions, common in patients after cardiac arrest [33,35]. Myoclonus that occurs early, as generalised, synchronous and stereotyped and lasts for more than 30 min is usually referred to as status myoclonus [35]. This is usually associated with worse outcomes [24,33,35], especially if the EEG is non-reactive. However, favourable outcome has been reported in patients with status myoclonus [36]. Myoclonus can be inhibited by sedation and neuromuscular blocking drugs.

Consistent with the literature, all patients with seizures except a single patient with myoclonus and a malignant rather than highly malignant EEG had unfavourable outcomes in the current study. Continuous EEG (cEEG) monitoring can be helpful in active treatment of status epilepticus. cEEG is labour-intensive and costly as it requires constant expert monitoring and real-time reporting, which can be difficult [35]. It can be used to monitor EEG patterns during administration of ASM [37]. The benefits are debatable, and a viable alternative is prolonged 1–3 or 6 h of cEEG monitoring or routine intermittent 30 min EEGs over a few days. Some centres may routinely use cEEG in all patients following cardiac arrest, but cEEG is rarely performed in our setting.

### 4.7. Benefits and Limitations of EEG

EEG is now a standard tool used to aid prognostication in patients with post-anoxic coma that provides useful information to influence decision making. However, the evaluation of EEG patterns in resuscitated and comatose patients after anoxic brain injury has its limitations. Just like clinical examination, the EEG is prone to interference from the initial treatment of patients including sedation and TH.

It is well known that EEG activity is affected by sedation [9,13,15,26,38] and can cause various changes in the EEG. The EEG is either markedly suppressed or will show burst suppression by sedative and anaesthetic drugs. But, it is not known if or how the sedation affects or helps with the prognostic value of malignant EEG patterns. So, interpreting such EEGs needs caution. Moreover, the neuronal activity would already be affected by the anoxic episode immediately post-resuscitation and is not stable. It could show any of the variety of patterns depending on the timing, after the anoxic episode, of the recording [13,39]. Early prognostication of neurological recovery is important given the ethical, family and economic burden.

In the early post-anoxic period, EEG changes are dynamic, so the timing of recording is important in interpreting EEG [8,11], and it emphasises the benefit of repeating this investigation. The effects of sedation and TH may combine as decreased body temperature prolongs the metabolism, particularly of sedation, causing difficulty in knowing if residual sedation is still affecting the patient [26] and, consequently, the EEG findings. Further, the interpretation of the EEG is complex and inclined to subjectivity. This is especially true of EEG recordings in ICU because of the variety of periodic and rhythmic patterns of uncertain clinical significance and the poor interrater agreement [15,22].

These issues of subjectivity may be overcome by the more recent development and utilisation of objective quantitative EEG analyses. For example, the application of machine learning models to a combination of quantitative EEG metrics such as entropy and clinical data may be beneficial in predicting the outcome of post-anoxic coma [1,3,32]. This highlights that EEG data cannot be considered in isolation and must be considered with other clinical data and patient co-morbidities to best predict outcomes.

### 4.8. EEG in the Context of Multimodal Prognostication

Current European guidelines [40] suggest incorporating a variety of assessments for neurological prognostication alongside EEG, including clinical assessment (Glasgow motor score, pupillary and corneal reflexes), SSEP, blood biomarkers (neurone-specific enolase, NSE) and brain imaging (CT or MRI). Our centre does not perform SSEP or have timely access to NSE so we cannot address these in the present study. Hence, we chose to focus solely on EEG for the purposes of this study but recognise this as a limitation. We do, however, report the number of scans showing diffuse hypoxic injury based on the reports in accordance with these guidelines, and there is a clear association with EEG findings: diffuse hypoxic injury was seen in 57% of those with highly malignant EEG, 15% of those with malignant EEG and none with benign EEG.

### 4.9. Future Work

Whilst all those with high malignant EEG did not survive, and all those with benign EEG did survive, the malignant EEG category warrants further study to understand how the other prognostic markers included in guidelines can help guide management in this group. Follow-up EEG may be particularly beneficial in this group.

A recent sub-study of the Targeted Temperature Management Trial 2 suggested that the specificity of the highly malignant EEG patterns in predicting poor neurological outcomes may be lower in clinical practice than initially thought [41]. The authors suggest combining the highly malignant EEG patterns with an unreactive background to improve sensitivity. However, all of patients with highly malignant EEG in our study did also have an unreactive background.

Finally, this study looks at the experience of a single centre in relation to published literature and only looks at EEG patterns. This may limit the applicability of our results to other populations or clinical settings, so further multi-centre studies are required. Such studies would enable the incorporation of other clinical variables, including age and etiology, in a multivariate analysis.

## 5. Conclusions

Cardiac arrest is common, and hypoxic–ischemic injury carries a high mortality. EEG recordings form a key part of a multimodal evaluation for prognosis. We describe the prognostic role of EEG in a well-characterised cohort of patients from our centre. Highly malignant EEG, seizures and myoclonus were associated with unfavourable outcome, while reactive EEG and improvement on follow-up were associated with better outcomes. Patients with malignant EEG patterns need to be looked at more critically, as it is noted that the outcome can be variable and is more likely to be favourable. In our study, an EEG pattern of diffusely slow or with reversed anterior/posterior gradient without clear spike-and-wave discharges was more favourable, a finding which requires further validation. Other prognostic markers including clinical assessment, biomarkers and neuroimaging are essential considerations. Accurate early prognostication of neurological recovery is important given the ethical, family and economic implications of treatment.

## Figures and Tables

**Table 1:** Patient demographics, clinical features and relationship to outcomes based on CPC.

Feature	Number	Favourable Outcomes (CPC 1–2)	Unfavourable Outcomes (CPC 3–5)
Total patients	81	18 (22%)	63 (78%)
Female	24/81 (30%)	6 (24%)	19 (76%)
Male	57/81 (70%)	12 (21%)	45 (79%)
Therapeutic hypothermia	56/81 (69%)	10 (18%)	46 (82%)
Seizures on EEG	7/81 (9%)	0 (0%)	7 (100%)
Myoclonus on EEG	18/81 (22%)	1 (6%)	17 (94%)
Reactivity on EEG	25/80 (31%)	16 (64%)	9 (36%)
Improved on follow-up EEG	9/40 (23%)	6 (67%)	3 (33%)

**Table 2:** Causes of cardiac arrest stated in medical charts.

Causes	Number	%
Ventricular fibrillation	21	26
Myocardial infarction	20	25
Pulseless electrical activity	18	22
Respiratory arrest	4	5
Airway obstruction	3	4
Pulmonary embolism	2	2
Sepsis	2	2
Aortic dissection	1	1
Haemorrhagic stroke	1	1
Unknown	9	11
**TOTAL**	**81**	**100**

**Table 3:** Indications given for EEG on requisition form.

Indication	Number	%
Decreased level of consciousness	46	57
Myoclonic jerks/twitching	18	22
Possible seizure post-cardiac arrest	14	17
Agitated, confused	3	4
**TOTAL**	**81**	**100**

**Table 4:** Specific EEG patterns (highly malignant, malignant and benign) and the relationship to survival and outcomes on CPC.

Group	# of Pts	EEG Pattern	Improvement (If Repeated)	Reactivity	Seizures or Myoclonus	Diffuse Hypoxic Changes on Imaging	Survival	CPC on Follow-Up
**Highly malignant (*n* = 46)**								
(i)	23	Suppressed with continuous periodic discharges	0/20 (0%)	0/21 (0%) 1 SIRPIDs	15 (65%)	12/22 (55%)	0 (0%)	5 (100%)
(ii)	11	Burst suppression with or without discharges	0/4 (0%)	0/11 (0%)	6 (55%)	5/9 (56%)	0 (0%)	4 and 5 (100%)
(iii)	12	Suppressed background without discharges	0/3 (0%)	0/10 (0%)	0 (0%)	9/10 (90%)	0 (0%)	5 (100%)
**Malignant (*n* = 33)**								
(iv)	11	Periodic or rhythmic patterns (abundant periodic discharges)	5/7 (71%)	7/11(64%)	2 (9%)	2/11 (18%)	5 (45%)	1–2 (*n* = 4, 36%) 3 (*n* = 1, 9%) 4–5 (*n* = 6, 55%)
(v)	22	Discontinuous background; low voltage; reversed anterior–posterior gradient	3/5 (60%)	16/22 (73%)	0 (0%)	3/22 (14%)	18 (82%)	1–2 (*n* = 12, 55%) 3 (*n* = 6, 27%) 4–5 (*n* = 4, 18%)
**Benign (*n* = 2)**								
(vi)	2	Normal	1/1 (100%)	2/2 (100%)	0 (0%)	0/2 (0%)	2 (100%)	1 (*n* = 2, 100%)

## Data Availability

The datasets presented in this article are not openly available because the research ethics approval does not permit the sharing of individual raw patient data. Requests to access the summary datasets should be directed to the corresponding author.
